# Real-world prevalence of microsatellite instability testing and related status in women with advanced endometrial cancer in Europe

**DOI:** 10.1007/s00404-024-07504-3

**Published:** 2024-04-18

**Authors:** Sneha S. Kelkar, Vimalanand S. Prabhu, Jingchuan Zhang, Yoscar M. Ogando, Kyle Roney, Rishi P. Verma, Nicola Miles, Christian Marth

**Affiliations:** 1 OPEN Health, Bethesda, MD USA; 2 Merck & Co., Inc., 126 East Lincoln Ave, P.O. Box 2000, Rahway, NJ 07065 USA; 3 Eisai Inc., Nutley, NJ USA; 4 M3 (EU) Ltd., Abingdon, UK; 5 Department of Obstetrics and Gynecology, Medical University Innsbruck, Innsbruck, Austria

**Keywords:** Biomarkers, Oncology, Gynecology, Recurrent, Mismatch repair, Retrospective

## Abstract

**Purpose:**

To assess the real-world prevalence of microsatellite instability (MSI)/mismatch repair (MMR) testing and related tumor status in recurrent/advanced endometrial cancer patients in Europe.

**Methods:**

Data were from two multi-center, retrospective patient chart review studies conducted in the United Kingdom, Germany, Italy, France and Spain: The Endometrial Cancer Health Outcomes-Europe-First-Line (ECHO-EU-1L) study and the ECHO-EU-Second-Line (ECHO-EU-2L) study. ECHO-EU-1L included recurrent/advanced endometrial cancer patients who received first-line systemic therapy between 1/JUN/2016 and 31/MAR/2020 after recurrent/advanced diagnosis. ECHO-EU-2L included patients with recurrent/advanced endometrial cancer who progressed between 1/JUN/2016 and 30/JUN/2019 following prior first-line systemic therapy. Data collected included patient demographics, MSI/MMR tumor testing and results, and clinical/treatment characteristics.

**Results:**

ECHO-EU-1L included 242 first-line patients and ECHO-EU-2L included 475 s-line patients. For all patients, median age at recurrent/advanced diagnosis was 69 years, roughly half had endometrioid carcinoma histology and over 75% had Stage IIIB-IV disease at initial diagnosis. The prevalence of MSI/MMR testing in the first-line and second-line cohorts was similar (36.4 and 34.9%, respectively). Among those tested, a majority had non-MSI-high/MMR proficient tumors (80.7 and 74.7% among first- and second-line patients, respectively). About 15% had MSI-high/MMR deficient tumors in both cohorts, and a few patients had discordant results (3.4 and 10.8% among first- and second-line patients, respectively).

**Conclusion:**

Prior to the approvals of biomarker-directed therapies for recurrent/advanced endometrial cancer patients in Europe, there were low MSI/MMR testing rates for these patients of just over one-third. Given the availability of biomarker-directed therapies, increased MSI/MMR testing may help inform treatment decisions for recurrent/advanced endometrial cancer patients in Europe.

## What does this study add to the clinical work

**Table:** 

This study provides historical real-world evidence on MSI/MMR testing rates and related tumor status in recurrent/advanced endometrial cancer patients in Europe who received first-line or second-line systemic therapy since mid-2016, prior to the approvals of biomarker-directed therapies. Study findings showed low MSI/MMR testing rates and a lack of consensus on MSI/MMR testing requirements and timing, regardless of line of therapy, in real-world European clinical practice.	

## Introduction

Endometrial cancer has seen a steady increase in incidence in the past few decades, particularly in developed countries [1]. In 2020, there were 417,367 incident cases and 97,370 deaths globally attributable to endometrial cancer, with Europe having the second highest cumulative incident cases and deaths, second only to Asia [2]. Although patients diagnosed with early-stage disease have good prognosis, for the 10–13% of patients who are diagnosed with advanced stage III and stage IV disease, 5-year survival is low (60 and 29% survival, respectively) [3, 4]. Furthermore, the 3-year recurrence rate for patients diagnosed with early stage disease is estimated at 6.5–7%, with varying overall 5-year survival depending on the extent of metastasis (17.5–64.8%) [5, 6].

Traditionally, treatment for endometrial cancer has consisted of a combination of surgery, radiotherapy, and/or chemotherapy, depending on disease stage and histology. For patients with recurrent or advanced disease who are not candidates for surgery or radiation therapy, chemotherapy with carboplatin and paclitaxel or hormonal therapy were recommended as the standard systemic therapies in the front-line, while there was no consensus on standard of care in later lines [7, 8].

Microsatellite instability (MSI), a molecular alteration due to a defective deoxyribonucleic acid (DNA) mismatch repair (MMR) system, has emerged as an actionable biomarker in solid tumors [9]. Recent approvals of treatments in Europe targeting patient tumor status have changed the treatment landscape for patients with recurrent/advanced endometrial cancer. In April 2021, the European Commission granted conditional approval for a programmed cell death protein 1 checkpoint inhibitor, dostarlimab, for MSI-high (MSI-H)/MMR deficient (dMMR) advanced endometrial cancer patients who progressed on a prior line of platinum therapy [10]. In April 2022, the European Commission approved pembrolizumab monotherapy for the treatment of MSI-H or dMMR tumors in adults with advanced or recurrent endometrial carcinoma who have disease progression on or following prior treatment with a platinum-containing therapy in any setting and who are not candidates for curative surgery or radiation [11]. In January 2022, the European Commission also approved a novel regimen regardless of patient MSI/MMR tumor status: pembrolizumab in combination with lenvatinib for the treatment of recurrent/advanced endometrial cancer in adults who have disease progression on or following prior treatment with a platinum-containing therapy in any setting and who are not candidates for curative surgery or radiation therapy [7]. The recommendations from the 2022 European Society of Medical Oncology guidelines were updated to include these newly approved regimens for patients whose disease progressed following prior chemotherapy. In December 2023, the European Commission also approved dostarlimab paired with carboplatin and paclitaxel in adults with dMMR/MSI-H primary advanced or recurrent endometrial cancer who are eligible to receive systemic treatment (including in front-line settings) [12]. Novel therapies continue to be investigated in several clinical trials for use with recurrent/advanced endometrial cancer patients with MSI-H/dMMR or non-MSI-H/MMR proficient (pMMR) tumors in first-line settings [13–15].

Despite the evolving landscape and rising importance of tumor biomarkers in treatment selection, real-world testing rates for MSI/MMR and related tumor status in patients with recurrent/advanced endometrial cancer in Europe are not well documented [16]. We conducted two retrospective real-world data studies, the Endometrial Cancer Health Outcomes-Europe-First-Line (ECHO-EU-1L) study and the ECHO-Europe-Second-Line (ECHO-EU-2L) study aimed at evaluating the real-world prevalence of MSI/MMR testing and related tumor status in recurrent/advanced endometrial cancer patients receiving first-line or second-line systemic therapies in Europe [17, 18].

## Methods

### Study design and eligibility criteria

Our studies were multi-center, retrospective patient medical chart reviews conducted in the United Kingdom, Germany, Italy, France and Spain. Geographically dispersed, random samples of endometrial cancer-treating oncologists (medical oncologist, gynecologic oncologist or clinical oncologist) were recruited separately from each country for each study. Oncologists provided data from eligible patients’ medical records and data were de-identified before analyses. The studies were similar with respect to their study design, data collected and analyses.

For ECHO-EU-1L, all female patients managed by the participating oncologists ≥ 18 years of age were eligible if they received a first-line systemic therapy between July 1, 2016 and March 31, 2020 after diagnosis of recurrent/advanced (stage III or IV) endometrial cancer.

For ECHO-EU-2L, all female patients managed by the participating oncologists ≥ 18 years of age diagnosed with inoperable recurrent/advanced endometrial cancer between July 1, 2016 and December 31, 2018 were eligible if they had received at least one systemic therapy after diagnosis and progressed between July 1, 2016 and June 30, 2019.

Patients were excluded from either study if they were enrolled in any endometrial cancer clinical trial during the study period or had any malignancy active within the 3 years prior to recurrent/advanced endometrial cancer diagnosis (except for locally curable cancers that had been cured). Patients were excluded from ECHO-EU-1L if they initiated first-line therapy with immunotherapy (as monotherapy or any combination therapy) or lenvatinib.

### Data collection and study measures

For both studies, patient data were entered by participating oncologists into electronic case report forms via a secure online portal. Patients were randomly selected among all eligible patients managed by the managing oncologist based on the first letter of their last name, as indicated by a random letter generator. Data collected included patient demographics, MSI/MMR tumor testing occurrence (polymerase chain reaction and immunohistochemistry) and related results including expression of MMR proteins (for those tested), and clinical/treatment characteristics.

Patients were categorized by MSI/MMR status as the following: non-MSI-H (including microsatellite stable [MSS] and MSI-low [MSI-L]) or pMMR; MSI-H or dMMR; or patients with discordant results (patients with test results indicating overlapping tumor status such as non-MSI-H with dMMR or MSI-H with pMMR).

### Statistical analyses

Categorical variables were summarized using percentage and count. Continuous variables were summarized using the summary statistics of mean and standard deviation or median and interquartile range, as appropriate. Results herein are presented separately for first-line and s-line recurrent/advanced endometrial cancer patients.

All statistical analyses were conducted using SAS version 9.4.

## Results

### Physician characteristics

Across all countries, a total of 57 and 103 physicians participated in ECHO-EU-1L study and ECHO-EU-2L study, respectively. In both studies, physicians were primarily medical oncologists (94.7% ECHO-EU-1L, 89.3% ECHO-EU-2L), and had been in practice for more than 10 years (78.9, 76.7%). Almost all physicians practiced in an urban setting (96.5, 96.1%) and were part of a hospital practice (91.2, 92.2%). Most physicians primarily practiced at an academic hospital (77.2, 75.7%), followed by community hospital (19.3, 22.3%) and private office (1.8, 2.9%).

### Demographic and clinical characteristics of first- and second-line recurrent/advanced endometrial cancer patients

ECHO-EU-1L included 242 eligible first-line recurrent/advanced endometrial cancer patients, including 49 from the United Kingdom, 49 from France, 48 from Germany, 48 from Italy and 48 from Spain. ECHO-EU-2L included 475 eligible second-line recurrent/advanced endometrial cancer patients, including 101 from the United Kingdom, 96 from France, 88 from Germany, 100 from Italy and 90 from Spain (Table 1).

**Table 1:** Demographics and clinical characteristics for recurrent or aEC patients on first-line and second-line systemic therapy

	EU 1L	EU 2L
	(N = 242)	(N = 475)
Age at diagnosis of recurrent or aEC (years)		
Median (Q1 to Q3)	69 (64 to 74)	69 (64 to 74)
Recurrent or advanced disease		
Recurrent	27 (11.2)	50 (10.5)
Advanced	215 (88.8)	425 (89.5)
Race^a^, N (%)		
White or Caucasian	183 (94.8)	366 (96.6)
Black or African/Caribbean-origin	2 (1.0)	6 (1.6)
Middle Eastern/North-African	2 (1.0)	4 (1.1)
Asian	6 (3.1)	3 (0.8)
Charlson Comorbidity Index		
Median (Q1 to Q3)	1 (0.0 to 2.0)	1 (0.0 to 2.0)
Staging at initial diagnosis, N (%)		
IA	3 (1.2)	7 (1.5)
IB	3 (1.2)	26 (5.5)
II	21 (8.7)	17 (3.6)
IIIA	16 (6.6)	51 (10.7)
IIIB	8 (3.3)	27 (5.7)
IIIC	39 (16.1)	56 (11.8)
IV	152 (62.8)	291 (61.3)
Histology at diagnosis, N (%)		
Endometrioid carcinoma	119 (49.2)	274 (57.7)
Clear cell carcinoma	44 (18.2)	75 (15.8)
Serous carcinoma	34 (14.0)	74 (15.6)
Mucinous carcinoma	19 (7.9)	11 (2.3)
Carcinosarcoma	13 (5.4)	18 (3.8)
Undifferentiated carcinoma/Mixed cell tumors	7 (2.9)	16 (3.4)
Squamous cell carcinoma	6 (2.5)	7 (1.5)
Metastatic site(s) at diagnosis^b^, N (%)		
Distant lymph nodes	92 (66.7)	168 (61.5)
Lung	71 (51.4)	141 (51.6)
Liver	68 (49.3)	106 (38.8)
Bone	17 (12.3)	27 (9.9)
Brain	3 (2.2)	1 (0.4)
Pancreas	2 (1.4)	1 (0.4)
Kidneys	1 (0.7)	1 (0.4)
Other	27 (19.6)	49 (17.9)
ECOG at the initiation of first-line therapy, N (%)		
0	27 (11.2)	
1	158 (65.3)	
2	52 (21.5)	
3	5 (2.1)	
ECOG at the initiation of second-line therapy, N (%)		
0		18 (3.8)
1		236 (49.7)
2		190 (40.0)
3		28 (5.9)
Unknown		3 (0.6)

*aEC* Advanced endometrial cancer; *ECOG* Eastern Cooperative Oncology Group; *N* Number; *Q1* First quartile; *Q3* Third quartile; *1L* First-line; *2L* Second-line

^a^Race data not available for France; percentages are calculated amongst patients from the other four countries

^b^Data on metastatic sites at diagnosis are out of N = 138 and N = 273 patients with known metastatic status for ECHO-EU-1L and ECHO-EU-2L, respectively

Demographic and clinical characteristics were similar across first- and second-line recurrent/advanced endometrial cancer patients. For all patients, median age at recurrent/advanced endometrial cancer diagnosis was 69 years, about 90% had advanced disease, and roughly 95% of patients were White/Caucasian. Race and ethnicity data were not collected in France due to legal restrictions. The most prevalent comorbidity across all patients was diabetes. A total of 49.2% of first-line and 57.7% of second-line patients had endometrioid carcinoma histology and over 75% of patients overall had Stage IIIB-IV disease at initial diagnosis. At initiation of first-line of therapy, 23.6% of first-line patients had poor Eastern Cooperative Oncology Group (ECOG) performance status of ≥ 2, while at initiation of second-line therapy, 45.9% of second-line patients had ECOG status of ≥ 2.

### Prevalence and timing of MSI/MMR testing among first- and second-line recurrent/advanced endometrial cancer patients

**Fig. 1:**
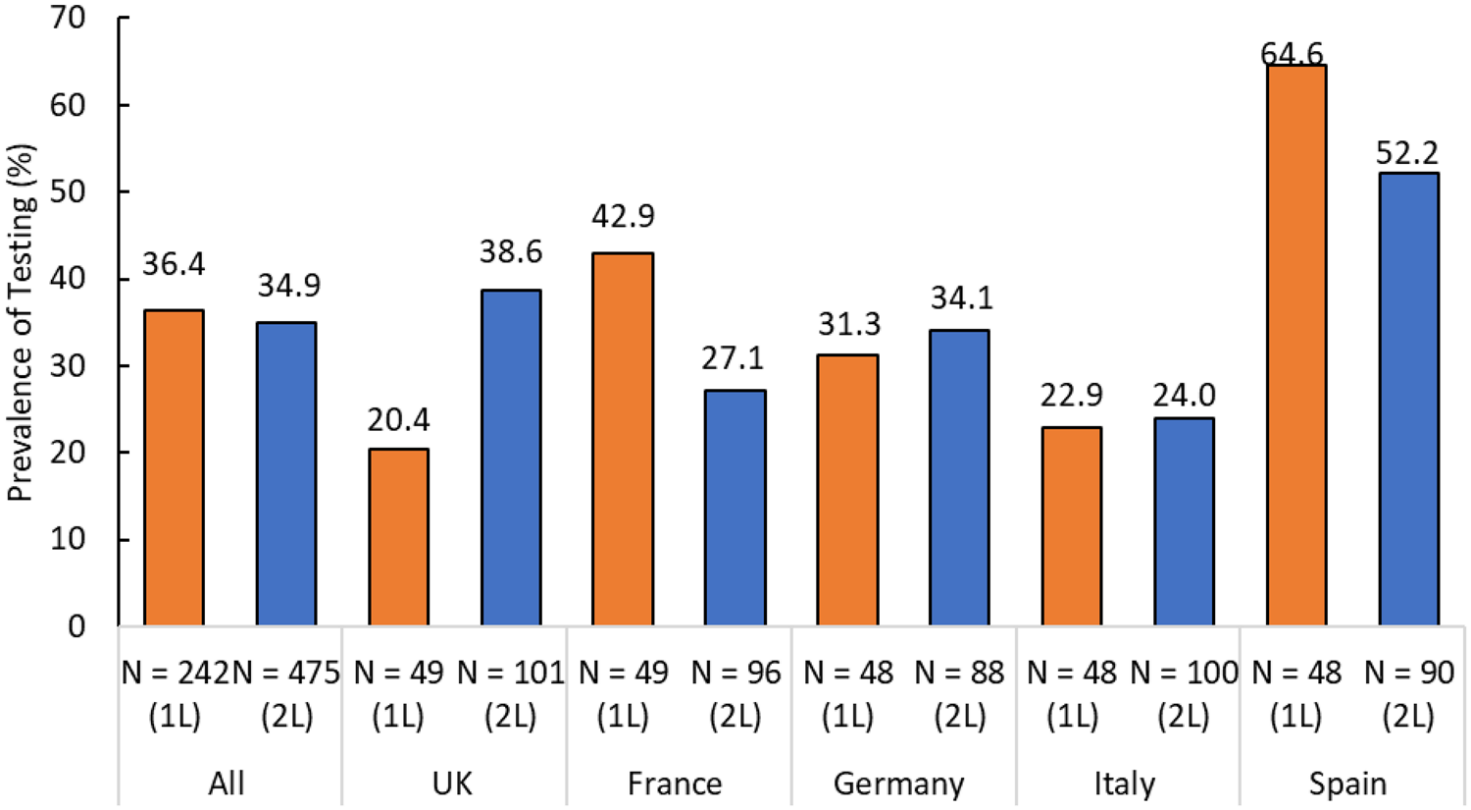
Prevalence of MSI/MMR testing by country among recurrent or aEC patients who initiated first-line therapy and in those who progressed on prior therapy and initiated second-line therapy. *aEC* Advanced endometrial cancer; *MSI* Microsatellite instability; *MMR* Mismatch repair; *N* Number; *UK* United Kingdom; *1L* First-line; *2L* Second-line

The prevalence of MSI/MMR testing using polymerase chain reaction and/or immunohistochemistry in the 242 first-line and 475 s-line recurrent/advanced endometrial cancer patients was similar [36.4% (n = 88) and 34.9% (n = 166), respectively] (Table 2 and Fig. 1). However, the prevalence of testing varied considerably across countries in both studies. Spain had the highest testing rates (64.6% among first-line and 52.2% among second-line patients), and lowest testing rates were observed in United Kingdom (among first-line patients, 20.4%) and Italy (among second-line patients, 24.0%) (Fig. 1).

**Table 2:** MSI/MMR testing pattern and results for recurrent or aEC patients on first-line and second-line systemic therapy

	EU 1L	EU 2L
	(N = 242)	(N = 475)
Any MSI/MMR testing (IHC or PCR), N (%)		
Not tested	154 (63.6)	309 (65.1)
Tested	88 (36.4)	166 (34.9)
Type of MSI/MMR testing, N (%)		
Only IHC tested	44 (50.0)	88 (53.0)
Only PCR tested	17 (19.3)	26 (15.7)
Both IHC and PCR tested	27 (30.7)	52 (31.3)
MSI/MMR status, N (%)		
Non-MSI-H/pMMR	71 (80.7)	124 (74.7)
MSI-H/dMMR	14 (15.9)	24 (14.5)
Discordant	3 (3.4)	18 (10.8)
Timing of first PCR or IHC test, N (%)		
Before 1L start	56 (63.6)	96 (57.8)
After 1L start but before 2L start	29 (33.0)	37 (22.3)
After 2L start	3 (3.4)	33 (19.9)
Any IHC testing for MMR status conducted, N (%)	71 (29.3)	140 (29.5)
Type of IHC/MMR tests used, N (%)		
NeoGenomics MMR panel	30 (42.3)	42 (30.0)
Whole-Exome sequencing	15 (21.1)	17 (12.1)
Caris tumor profiling	5 (7.0)	13 (9.3)
Impact Genetics somatic tumor MMR sequencing and deletion/duplication	2 (2.8)	18 (12.9)
Other	19 (26.8)	52 (37.1)
Result of IHC test, N (%)	59 (83.1)	103 (73.6)
pMMR	12 (16.9)	37 (26.4)
dMMR		
dMMR expression, N (%)		
MLH1	7 (58.3)	23 (62.2)
PMS2	7 (58.3)	11 (29.7)
MSH6	3 (25.0)	9 (24.3)
MSH2	1 (8.3)	15 (40.5)
Any PCR testing for MSI status conducted, N (%)	44 (18.2)	78 (16.4)
Type of PCR tests used, N (%)	17 (38.6)	29 (37.2)
Foundation One CDx	13 (29.5)	18 (23.1)
Whole-Exome sequencing	6 (13.6)	7 (9.0)
Caris tumor profiling	2 (4.5)	11 (14.1)
TrueMark MSI Assay	6 (13.6)	14 (17.9)
Other		
Result of PCR/MSI test, N (%)		
MSS	35 (79.5)	42 (53.8)
MSI—L	2 (4.5)	19 (24.4)
MSI—H	7 (15.9)	17 (21.8)

*aEC* Advanced endometrial cancer; *dMMR* Mismatch repair deficient; *IHC* Immunohistochemistry; *MLH1* MutL homolog 1; *MMR* Mismatch repair; *MSH2* MutS homolog 2; *MSH6* MutS homolog 6; *MSI* Microsatellite instability; *MSI-H* Microsatellite instability high; *MSI-L* Microsatellite instability low; *MSS* Microsatellite stable; *N* Number; *PCR* Polymerase chain reaction; *pMMR* Mismatch repair proficient; *PMS2* PMS1 homolog 2 mismatch repair system component; *1L* First-line; *2L* Second-line

Across both the studies, immunohistochemistry was the more commonly administered test to assess the tumor status, with NeoGenomics MMR panel being one of the most common test types. Among the 88 first-line patients who were tested, half (50.0%) of them received only an immunohistochemistry test for MMR status, 19.3% received only a polymerase chain reaction test for MSI status, and 30.7% received both tests. Among the 166 s-line patients who were tested, over half (53.0%) of the patients received only an immunohistochemistry test for MMR status, 15.7% received only a polymerase chain reaction test for MSI status, and 31.3% received both tests.

In the ECHO-EU-1L study, a majority of patients were tested prior to initiation of first-line therapy (63.6%) but 33.0% of patients were tested after discontinuation of first-line but prior to initiation of second-line therapy. There was considerable variation among countries as 100% of patients were tested prior to initiation of first-line in the United Kingdom compared with 45.5% in Italy. In the ECHO-EU-2L study, 57.8% were tested prior to initiation of first-line therapy; a total of 80.1% patients were tested prior to the initiation of second-line therapy, and there was less variation across countries (58.3% [Italy]–84.6% [UK and France]).

### MSI/MMR testing results among first- and second-line recurrent/advanced endometrial cancer patients

Among the 88 first-line patients tested, 71 (80.7%) had non-MSI-H/pMMR tumors, 14 (15.9%) had MSI-H/dMMR tumors, and 3 (3.4%) had discordant results. Among the 166 s-line patients tested, 124 (74.7%) had non-MSI-H/pMMR tumors, 24 (14.5%) had MSI-H/dMMR tumors, and 18 (10.8%) had discordant results. Results varied across countries, with the highest and lowest prevalence of non-MSI-high/pMMR tumors in the United Kingdom (90.0%) and Germany (53.3%) among first-line patients, and France (88.5%) and United Kingdom (56.4%) among second-line patients. These variations are likely due to the small number of patients who received testing overall and in each country.

Among first-line patients with dMMR status, loss of expression of MMR proteins was most commonly as MLH1 (58.3%), PMS2 (58.3%), MSH6 (25.0%), and MSH2 (8.3%). Among second-line patients with dMMR status, loss of expression of MMR proteins was most commonly as MLH1 (62.2%), MSH2 (40.5%), PMS2 (29.7%), and MSH6 (24.3%).

### Trends in MSI/MMR testing among first- and second-line recurrent/advanced endometrial cancer patients

**Fig. 2:**
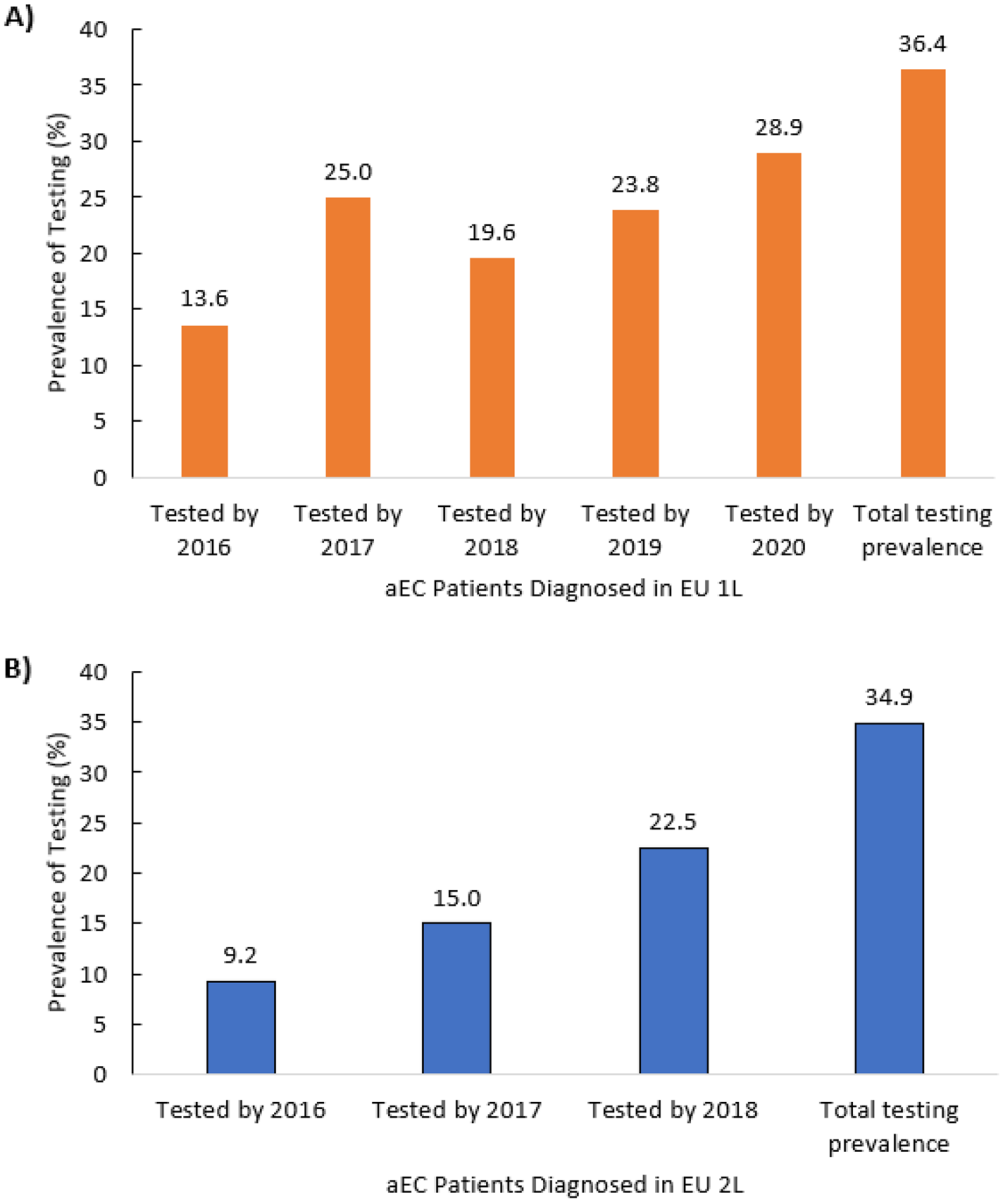
Prevalence of MSI/MMR testing by year of diagnosis among recurrent or aEC patients who initiated first-line therapy (**A**) and in those who progressed on prior therapy and initiated second-line therapy (**B**). *aEC* Advanced endometrial cancer; *MSI* Microsatellite instability; *MMR* Mismatch repair; *1L* First-line; *2L* Second-line

For first-line patients, the cumulative prevalence of MSI/MMR testing in patients diagnosed and tested by the year 2016 was 13.6% (n = 3). It increased to 25.0% (n = 15) in those diagnosed and tested by 2017, decreased to 19.6% (n = 20) by 2018, and increased over the next 2 years to 28.9% (n = 70) by 2020. All patients were diagnosed by 2020, but 7.5% (n = 18) were tested post-2020 until end of follow-up period. For second-line patients, the cumulative prevalence of MSI/MMR testing in patients diagnosed and tested by the year 2016 was 9.2% (n = 11). It increased to 15.0% (n = 40) in those diagnosed and tested by 2017, and 22.5% (n = 107) by 2018. All patients were diagnosed by 2018, but 12.4% (n = 59) were tested post-2018 until end of follow-up period (Fig. 2).

**Fig. 3:**
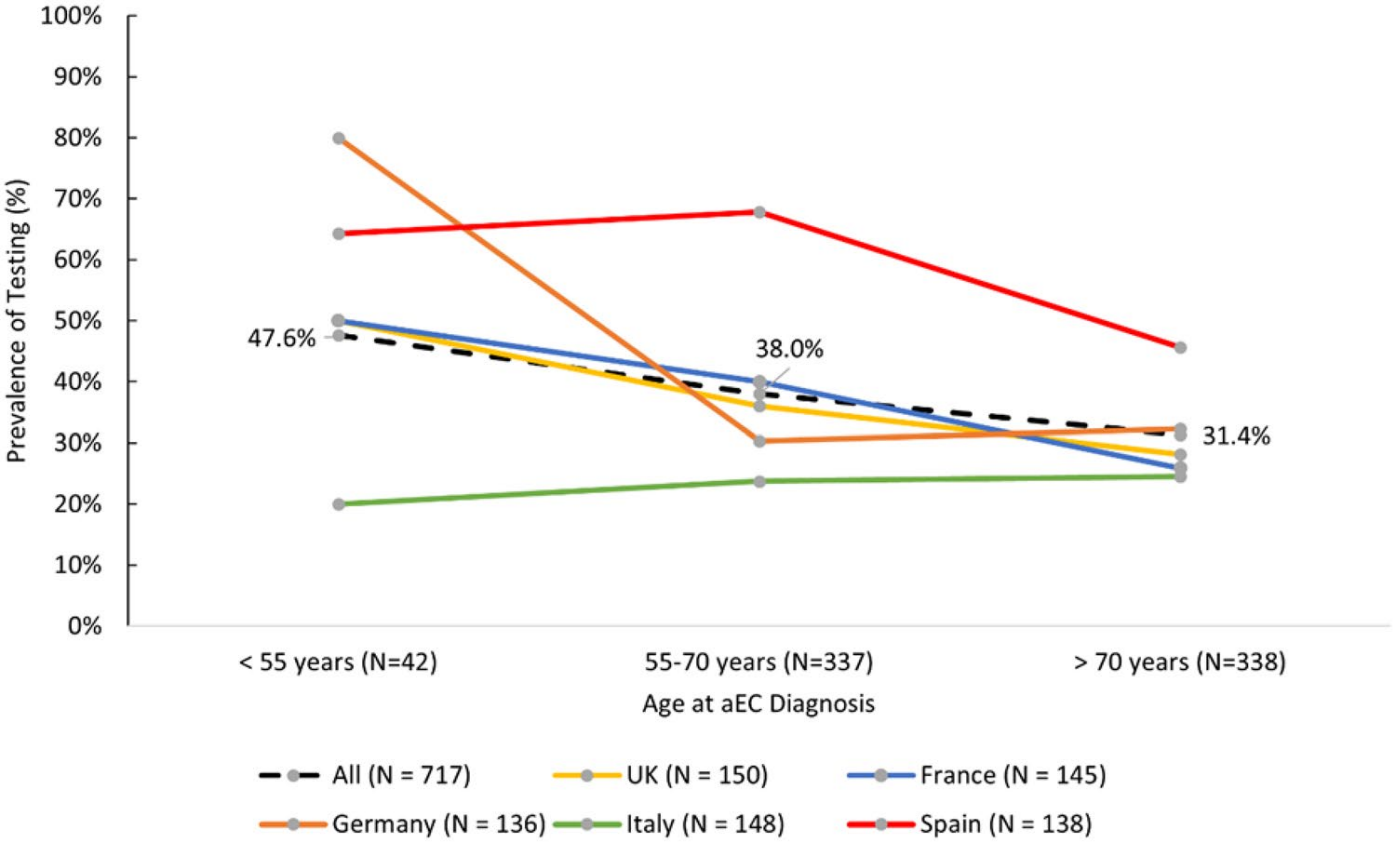
Prevalence of MSI/MMR testing by age at diagnosis and by country among recurrent or aEC patients who initiated first-line therapy and in those who progressed on prior therapy and initiated second-line (combined 1L and 2L populations). *aEC* Advanced endometrial cancer; *MSI* Microsatellite instability; *MMR* Mismatch repair; *N* Number; *UK* United Kingdom; *1L* First-line; *2L* Second-line

The prevalence of MSI/MMR testing was higher in younger patients. In a combined estimate among all patients from both studies, 47.6% of patients diagnosed under 55 years of age were tested, which decreased in patients who were diagnosed between 55 and 70 years of age (38.0%) and at 70 years of age or older (31.4%) (Fig. 3).

## Discussion

### Summary of main results

To our knowledge, this is the first comprehensive real-world assessment of MSI/MMR testing rates and related tumor status in first-line and second-line (following prior systemic therapy) recurrent/advanced endometrial cancer patients in Europe, using data from two retrospective, multi-country observational studies. Our studies provide information on the historic MSI/MMR testing patterns for patients that were diagnosed and treated during 2016–2020. Data were provided by geographically dispersed physicians on broad recurrent/advanced endometrial cancer patient populations.

Overall MSI/MMR testing prevalence in Europe during 2016–2020 was low at roughly 35% for both first-line and second-line recurrent/advanced endometrial cancer patients and varied considerably by country. It was highest in Italy for all patients, and lowest in the United Kingdom among first-line patients and Spain among second-line patients. Among patients who received an MSI/MMR test, roughly one-third and one-fifth of patients were not tested until after initiation of first- or second-line therapy, respectively. Our studies indicate that patient age at diagnosis of recurrent/advanced endometrial cancer may play a role in whether a patient receives MSI/MMR tumor testing. The overall prevalence of MSI/MMR testing in Europe decreased with patient age, regardless of line of therapy, although there was a marked variation across countries. Our studies also found that there was a gradual increase in the prevalence of MSI/MMR testing from 2016 to 2019/2020.

A majority of patients tested had non-MSI-H/pMMR tumors (roughly 81 and 75% among first- and second-line patients, respectively), about 15% had MSI-H/dMMR tumors (among both patient cohorts), and the remaining patients reported discordant results.

### Results in the context of published literature

In a retrospective study conducted in the United States (US), the overall MSI/MMR testing rate in recurrent/advanced endometrial cancer patients who progressed following prior systemic therapy from mid-2016 to mid-2019 was 92.2%, and among tested patients, 62.0% had non-MSI-H/pMMR tumors while 38.0% had MSI-H/dMMR tumors [19]. The higher testing rate in the US could be attributable to the earlier approval and availability of MSI/MMR biomarker-directed therapies in the US, as well as screening for Lynch syndrome through universal tumor testing for all EC patients, based on recommendation by the Society for Gynecology Oncology (SGO) in 2014 [20]. The prevalence of MSI/MMR tumor status found in our studies is also corroborated by a global multi-country meta-analysis which found an overall prevalence of MSI-H of 17.6% (95% confidence interval (CI): 9.6–27.2%) [9].

### Strengths and weaknesses

Our studies have several limitations. First, study results are subject to extraction or measurement error. Multiple efforts before and after data extraction were made to ensure data accuracy and consistency and to minimize error. Second, the data extracted were limited by information available in patients’ medical charts, which may lack documentation of tumor testing. Third, given the nature of chart review studies which require physician consent to participate, these studies are subject to potential physician and patient selection bias. Broad physician samples, though mostly from academic practice settings, were recruited and a random patient selection method was implemented to minimize the potential selection bias and improve generalizability of the results across Europe. Our studies also have several strengths. First, they integrated data from five European countries with random samples of eligible patients selected from all geographic regions of the included countries; the study samples represented a large and broad patient population, regardless of demographics and clinical characteristics, supporting the generalizability of our study results. Second, the study periods allowed for sufficient follow-up for the collection of testing information.

### Implications for practice and future research

Findings from our studies indicate that there has been a lack of consensus on MSI/MMR testing requirements and timing, regardless of line of therapy, in real-world European clinical practice. Moreover, there was a rising but slow uptake of MSI/MMR testing in real-world European clinical practice. This rate is expected to increase after 2021 with the current approvals of novel therapies for recurrent/advanced endometrial cancer patients based on MSI/MMR tumor status. Moreover, the 2022 guidelines by the European Society for Medical Oncology now recommend MMR testing for all endometrial cancer patients regardless of histology. In addition to MSI/MMR, other molecular classifications of endometrial cancer such as p53 or POLE mutations may also have prognostic significance [21]. Thus, tumor biomarkers appear to be increasing in importance for the treatment of patients with endometrial cancer. Given the recent availability of biomarker-directed therapies since 2021, increased MSI/MMR testing may help better inform treatment decisions for first-line and pre-treated second-line recurrent/advanced endometrial cancer patients in Europe. It would be beneficial to monitor any trends in testing since the updated guidelines and availability of biomarker-directed therapies [7].

## Conclusion

Our studies provide real-world MSI/MMR testing rates and related tumor status in recurrent/advanced endometrial cancer patients in Europe who received first-line or second-line systemic therapy since mid-2016. Our study results showed low MSI/MMR testing rates of just over one-third in Europe among patients with recurrent/advanced endometrial cancer, with a slight increase from 2016 to 2020 and with considerable variation in testing rate and practices among countries.

## Data Availability

In accordance with the journal’s guidelines, we will provide our data for independent analysis by a selected team by the Editorial Team for the purposes of additional data analysis or for the reproducibility of this study in other centers if such is requested.
