# Correction: Real-world prevalence of microsatellite instability testing and related status in women with advanced endometrial cancer in Europe

**DOI:** 10.1007/s00404-024-07620-0

**Published:** 2024-07-13

**Authors:** Sneha S. Kelkar, Vimalanand S. Prabhu, Jingchuan Zhang, Yoscar M. Ogando, Kyle Roney, Rishi P. Verma, Nicola Miles, Christian Marth

**Affiliations:** 1 OPEN Health, Bethesda, MD USA; 2 Merck & Co., Inc., 126 East Lincoln Ave, P.O. Box 2000, Rahway, NJ 07065 USA; 3 Eisai Inc., Nutley, NJ USA; 4 M3 (EU) Ltd., Abingdon, UK; 5 Department of Obstetrics and Gynecology, Medical University Innsbruck, Innsbruck, Austria

**Correction: Archives of Gynecology and Obstetrics (2024) 309:2833–2841** 10.1007/s00404-024-07504-3

The copyright holder for this article was incorrectly given as “© The Author(s) 2024” but should have been "© Christian Marth, OPEN Health, Eisai, M3 EU Ltd, Merck & Co., Inc., Rahway, NJ, USA and its affiliates 2024".

The original article has been corrected.

